# Basal Cell Nevus Syndrome Treated With Laser Therapy: Novel Approach for Young Adults

**DOI:** 10.1111/ijd.17845

**Published:** 2025-05-27

**Authors:** Giulia Briatico, Camila Scharf, Eugenia Veronica Di Brizzi, Gabriella Brancaccio, Nikola Drmac

**Affiliations:** ^1^ University of Campania Naples Italy; ^2^ European Institute of Dermatology Milan Italy

**Keywords:** dermato‐oncology, laser treatment, skin cancer, basal cell nevus syndrome, basal cell carcinoma, Gorlin‐Goltz syndrome

Basal cell nevus syndrome (BCNS), also known as Gorlin‐Goltz syndrome, is a rare inherited disorder with an autosomal dominant pattern that causes early‐onset basal cell carcinomas (BCCs). Its associated features include keratocysts, palmoplantar pits, falx cerebri calcification, skeletal abnormalities, frontal bossing, fibromas (in the heart and ovaries), medulloblastoma, glaucoma, and cleft lip/palate [1]. The syndrome arises from an overactive Sonic hedgehog signaling pathway, promoting tumor growth.

Standard treatments encompass surgery, Mohs surgery, electrodessication, curettage, photodynamic therapy, cryotherapy, radiotherapy, and topical agents such as imiquimod or 5‐fluorouracil. Despite their high efficacy, these methods often lead to scarring, which can affect patients' psychosocial well‐being. The European Association of Dermato‐Oncology (EADO) guideline [2] recommends treating multiple BCCs in BCNS patients with hedgehog pathway inhibitors (sonidegib, vismodegib), shown to reduce the size of existing BCCs and the rate of new surgically eligible BCCs. However, side effects like muscle cramps, dysgeusia, and weight loss, as well as contraindications in pregnancy, restrict their use in young adults. The management of BCNS remains a topic of debate, necessitating a multidisciplinary approach.

Laser therapy has emerged as a promising alternative, with studies supporting the pulsed dye laser (PDL, 595 nm) [3] and the neodymium‐doped yttrium aluminium garnet laser (Nd:YAG, 1064 nm) [4].

A 36‐year‐old woman with genetically confirmed BCNS, diagnosed 15 years earlier, presented in December 2022. She had undergone multiple excisions but refused systemic treatment due to psychological distress and her desire for pregnancy. After signing the informed consent, the proposed treatment included PDL for superficial BCCs and Nd:YAG for nodular ones.

Treatment parameters were based on prior studies:

- PDL (VBeam, Candela Corporation): 7 mm, 0.45 ms, 13 J, Dynamic Cooling Device (DCD) off, treating with a 3 mm margin.
- Nd:YAG (Candela Corporation): 6 mm, 170 J, 3 ms, DCD off.

Each lesion required 1 to 3 treatments, with sessions every 2 months for a total of six sessions over 12 months. The treatment endpoint was achieving gray‐purple purpura after each session, while dermoscopy assessed the absence of BCCs during follow‐up (Figures 1 and 2). PDL caused mild, short‐term pain, whereas Nd:YAG needed local anesthesia. Side effects included erythema, swelling, and crusting. Lesions healed within 2 weeks without infection or bleeding. After 1 year, no scarring was observed; however, diffuse depigmentation did occur. The patient was satisfied with the cosmetic outcomes.

**FIGURE 1:**
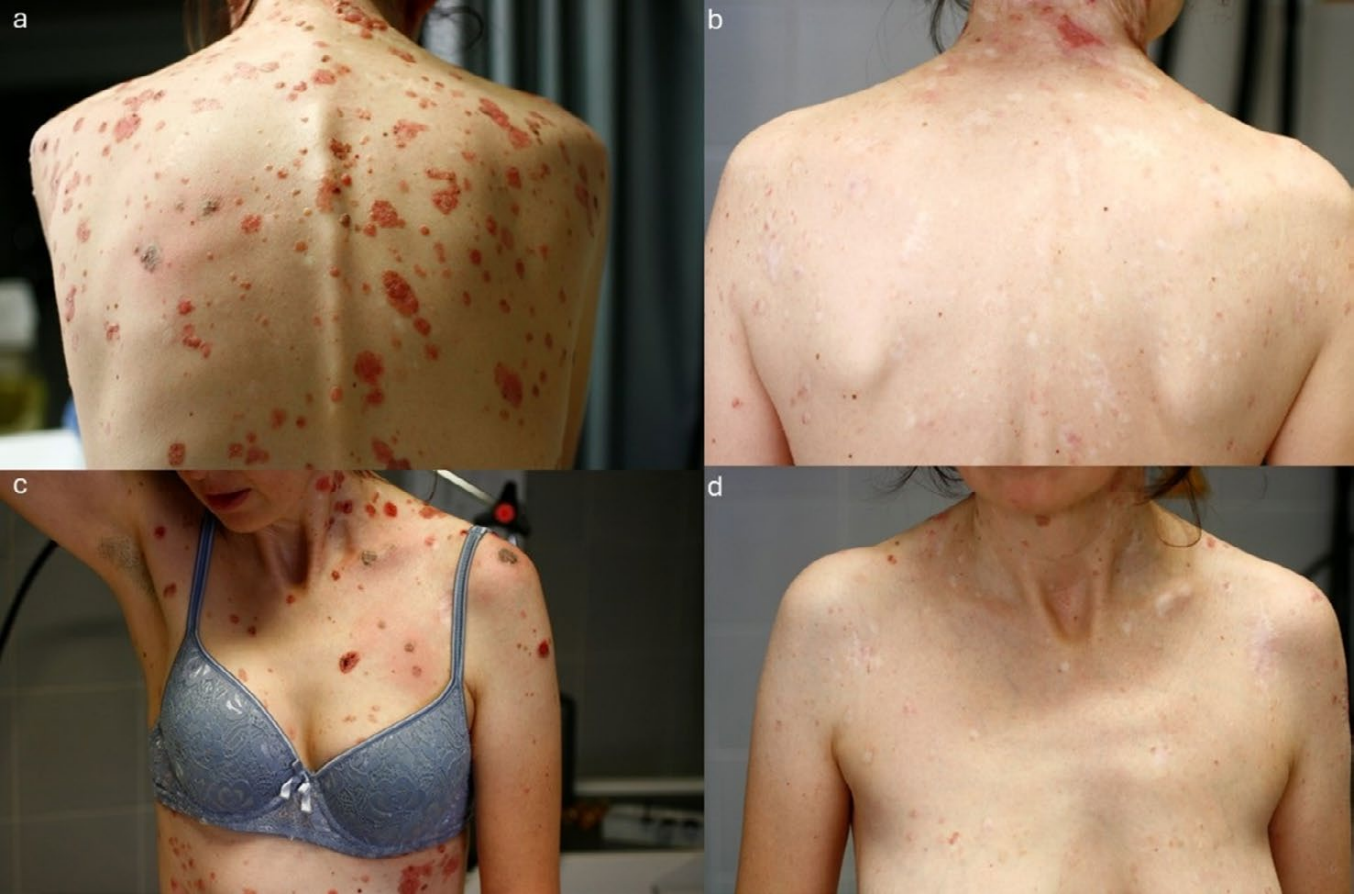
Images taken before (a–c) and after (b–d) 1 year of treatment.

**FIGURE 2:**
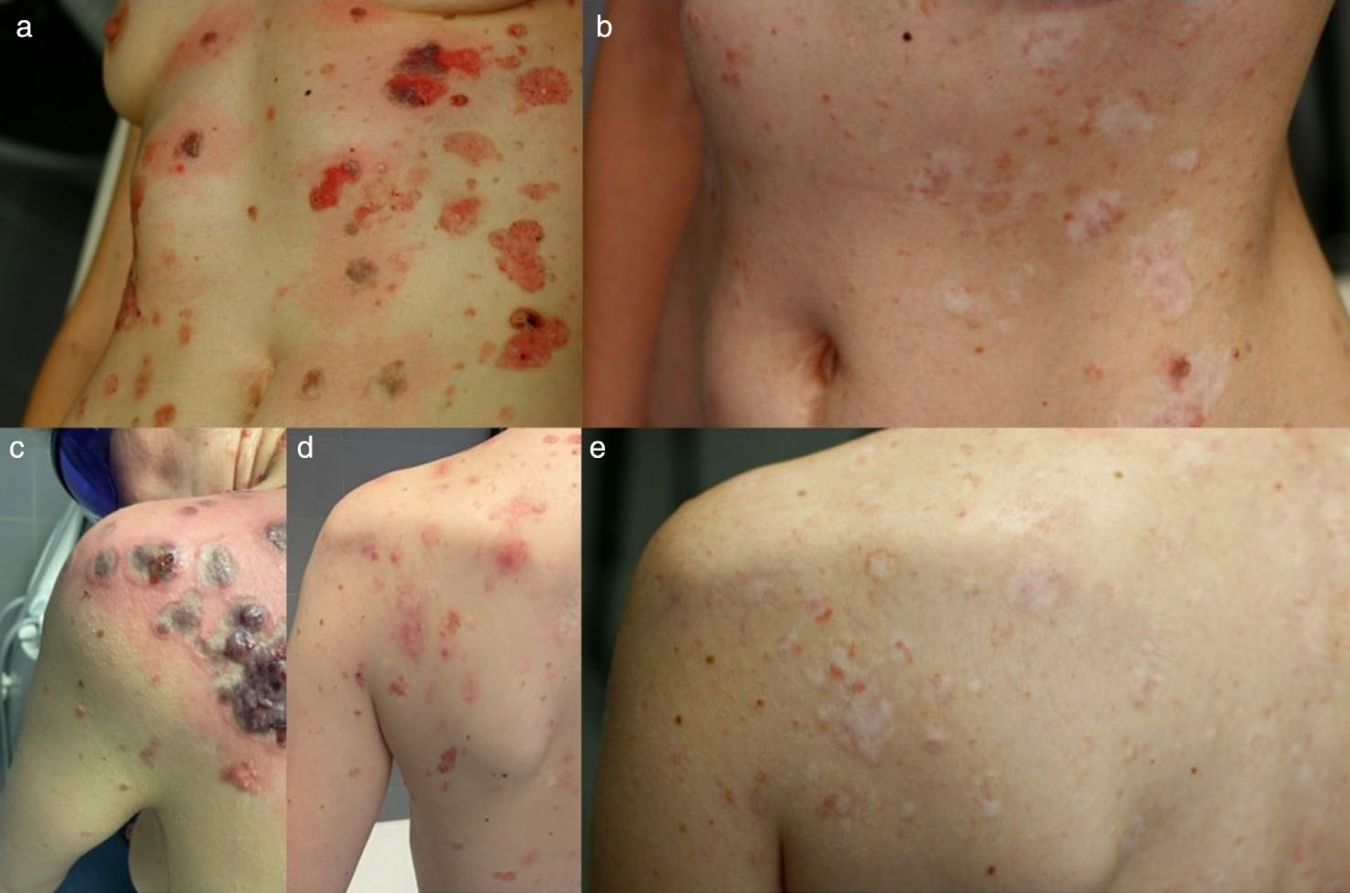
Before (a) and after (b). In (c), the dusky purpura is shown immediately following Nd:YAG treatment. In (d), it appears after 2 months, and in (e), after 1 year from the start of treatment.

This case highlights the necessity of effective non‐invasive treatments for young BCNS patients concerned about aesthetics. Carbon dioxide (CO_2_) laser ablation was not proposed, as it showed no improved cosmetic outcomes compared to cryotherapy. Additionally, it was not recommended in a systematic review of laser treatments for nonmelanoma skin cancer due to its lower effectiveness compared to Nd:YAG [5].

PDL, commonly used for vascular lesions, may also treat BCCs through an antiangiogenic effect. It disrupts the tumor's blood supply and reduces transforming growth factor‐beta (TGF‐β) expression, fibroblast proliferation, and collagen type III deposition—mechanisms also involved in keloid regression. Nd:YAG, which targets deep dermal lesions, is more effective for nodular BCCs due to its superior penetration depth.

In conclusion, PDL and Nd:YAG lasers present a valuable alternative to surgery for BCNS, offering favorable cosmetic results while postponing systemic therapy. This approach is especially beneficial for patients with aesthetic concerns but could also apply to those with disabilities, coagulopathy, or surgical contraindications. Further large‐scale studies are necessary to validate these findings.

## Conflicts of Interest

The authors declare no conflicts of interest.
